# Novel Class of Potential Therapeutics that Target Ricin Retrograde Translocation

**DOI:** 10.3390/toxins6010033

**Published:** 2013-12-23

**Authors:** Veronika Redmann, Thomas Gardner, Zerlina Lau, Keita Morohashi, Dan Felsenfeld, Domenico Tortorella

**Affiliations:** 1 Icahn School of Medicine at Mount Sinai, Department of Microbiology, One Gustave L. Levy Place, New York, NY 10029, USA; E-Mails: vredmann@path.wustl.edu (V.R.); thomas.gardner@mssm.edu (T.G.); 2 Icahn School of Medicine at Mount Sinai, Integrated Screening Core, Experimental Therapeutics Institute, One Gustave L. Levy Place, New York, NY 10029, USA; E-Mails: wingshan.lau@mssm.edu (Z.L.); Keita.morohashi@mssm.edu (K.M.); dan.felsenfeld@mssm.edu (D.F.)

**Keywords:** ricin toxin, small molecule inhibitors, high-content screen, retrograde translocation, stabilization, dislocation, egfp, ribosome-inactivating protein

## Abstract

Ricin toxin, an A-B toxin from *Ricinus communis*, induces cell death through the inhibition of protein synthesis. The toxin binds to the cell surface via its B chain (RTB) followed by its retrograde trafficking through intracellular compartments to the ER where the A chain (RTA) is transported across the membrane and into the cytosol. Ricin A chain is transported across the ER membrane utilizing cellular proteins involved in the disposal of aberrant ER proteins by a process referred to as retrograde translocation. Given the current lack of therapeutics against ricin intoxication, we developed a high-content screen using an enzymatically attenuated RTA chimera engineered with a carboxy-terminal enhanced green fluorescent protein (RTA_E177Q_egfp) to identify compounds that target RTA retrograde translocation. Stabilizing RTA_E177Q_egfp through the inclusion of proteasome inhibitor produced fluorescent peri-nuclear granules. Quantitative analysis of the fluorescent granules provided the basis to discover compounds from a small chemical library (2080 compounds) with known bioactive properties. Strikingly, the screen found compounds that stabilized RTA molecules within the cell and several compounds limited the ability of wild type RTA to suppress protein synthesis. Collectively, a robust high-content screen was developed to discover novel compounds that stabilize intracellular ricin and limit ricin intoxication.

## 1. Introduction

Ricin toxin is a member of the A-B family of toxins, which also includes cholera toxin, diphtheria toxin, shiga toxin, *Pseudomonas* exotoxin A and pertussis toxin [1]. Ricin toxin is a type-II ribosome inactivating protein, or RIP. Type II RIPs act upon the ribosome by depurinating an adenine residue in the region of the 28S rRNA termed the sarcin-ricin loop, thereby halting translation [2,3]. Ricin toxin enters the cell through endocytosis following interaction of the B subunit with cell surface glycolipids and glycoproteins. It traffics in a retrograde fashion through the trans-Golgi network and Golgi apparatus towards the endoplasmic reticulum (ER), gets transported across the ER membrane and eventually acts on its substrate, the ribosome, in the cytoplasm [4]. 

A critical step in movement of the RTA subunit towards its substrate in the cytoplasm is retrograde translocation across the ER membrane. In order to achieve retrograde translocation the RTA molecule interacts with cellular factors of the ER associated degradation pathway: Hrd1p, PDILT, ERO1L, DERL1, 2 and 3, UFD1L, NPLOC4, the Sec61p translocon, Hsc70, Hsp90 and the Rpt5 proteasome subunit [5,6,7,8,9]. Collectively, these studies suggest that RTA likely undergoes an unfolding step prior to dislocation and a refolding event following dislocation. The toxin would then proceed to inactivate ribosomes. 

Quality control in the ER directs terminally misfolded proteins for retrograde translocation from the ER and into the cytosol where they are degraded by the proteasome [10,11]. Misfolded proteins trigger ER stress sensors IRE1, PERK, and ATF6 that work to alleviate the stress by reducing translation levels and activating the transcription of chaperones to resolve the increased demand for folding assistance [12,13] The retrograde translocation and degradation of misfolded proteins is associated with disease states such as cystic fibrosis and emphysema, in which mutant forms cannot fold properly [14,15]. The ERAD pathway is utilized by cholera toxin, shiga toxin, and *Pseudomonas* exotoxin A to cross the ER membrane and by viruses such as HCMV, HSV-1, murine γ-herpesvirus 68, HIV, hepatitis B virus and SV40 to evade the immune system or increase productive infection [4,12,16]. Thus, ricin toxin has co-opted a cellular process to effectively gain access to the cytosol to inhibit protein synthesis [6,8,9].

Ricin toxin is a category B priority agent derived from the plant *Ricinus communis*, a common plant found in many areas of the world. The need to identify anti-ricin therapeutics is critical given properties that favor ricin’s potential use as a bioweapon: the plant’s broad ecological distribution and lack of treatment options following intoxication via inhalation or ingestion. Current anti-ricin efforts in high throughput screening have focused on blocking the enzymatic activity of the RTA subunit and intracellular trafficking events [17]. Our efforts in this study were directed towards blocking the retrograde translocation step in RTA trafficking by utilizing a human-cell based system we developed to study ricin transport across the ER membrane [18]. By generating a RTA chimera consisting of RTA and an egfp molecule, it was possible to visualize by fluorescent confocal microscopy the stabilization of RTA molecules as distinct peri-nuclear localized granules upon the treatment of proteasome inhibitor. We used the Granule Average Intensity to quantify the effect of individual compounds from a bioactive compound library on RTA_E177Q_egfp stabilization. These novel compounds stabilized the enzymatically attenuated RTA_E177D_ mutant and limited wild type RTA to inhibit protein synthesis and cytotoxicity. Collectively, the data supports a model that ER-localized RTA is a target for anti-ricin therapeutics.

## 2. Results and Discussion

### 2.1. RTA_E177Q_egfp Molecules Are Stabilized by Inclusion of Proteasome Inhibitor

**Figure 1:**
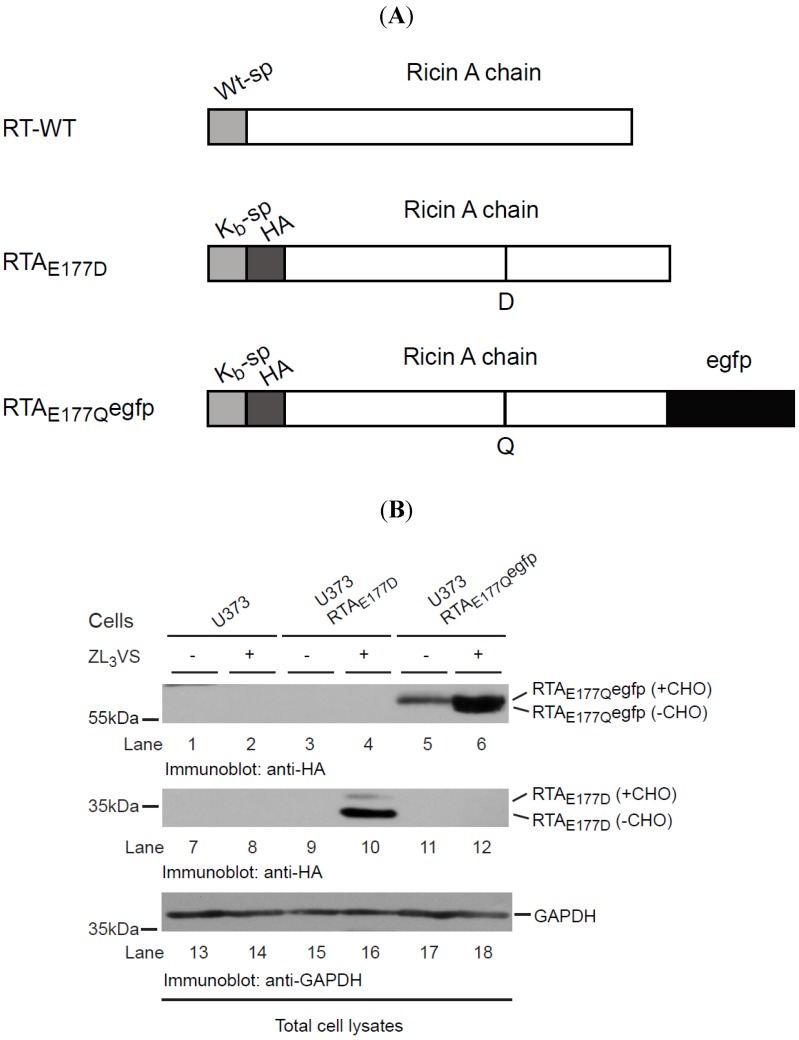
RTA_E177Q_egfp molecules are stabilized by proteasome inhibition. (**A**) Schematic diagram of wild type RTA (RTA-WT) and enzymatically-defective constructs (RTA_E177D_ and RTA_E177Q_egfp); (**B**) U373 cells and U373-RTA_E177D_- and U373-RTA_E177Q_egfp-expressing cells treated without or with ZL_3_VS (3 µM) were subjected to immunoblot analysis for RTA_E177Q_egfp (lanes 1–6), RTA_E177D_ (lanes 7–12), and GAPDH (lanes 13–18). RTA polypeptides and molecular weight markers are indicated.

Ricin A chain is transported across the ER membrane to the cytoplasm where it inhibits protein translation through the inactivation of ribosomes [19]. We previously established a human cell-based model to study the molecular requirements of RTA retrograde translocation using a catalytically inactive toxin because the wild type RTA inhibited protein synthesis [18]. Utilizing this cell-based RTA assay, we planned to develop a high-throughput screen to identify cell-permeable compounds that stabilize RTA within intracellular compartments, preventing its access to the ribosome and thus limiting ricin intoxication. To that end, a chimeric RTA was generated comprised of an enzymatically-attenuated RTA molecule (RTA_E177Q_) fused to an enhanced green fluorescent protein (egfp) (RTA_E177Q_egfp) (Figure 1A). Our initial experiments examined the stability of the chimeric RTA_E177Q_egfp molecule expressed in human U373 cells (U373-RTA_E177Q_egfp). U373 cells, U373-RTA_E177D_ cells, and U373-RTA_E177Q_egfp cells were treated for 16 h with ZL_3_VS (3 μM) and subjected to immunoblot analysis (Figure 1B). Stabilization of RTA molecules in the ER upon inhibiting proteasome activity is a result of blocking its degradation and thus affecting the retrograde translocation event [7,18]. As expected, proteasome inhibitor treated U373-RTA_E177D_ cells induced the accumulation of two polypeptides consisting of glycosylated and deglycosylated RTA proteins (Figure 1B, lane 10). Consistent with this result, proteasome inhibitor treatment caused an increase in RTA_E177Q_egfp polypeptides consistent with glycosylated and deglycosylated polypeptides (Figure 1B, lane 6 and Supplemental Figure 1). The observation of diverse amounts of glycosylated and deglycosylated of RTA_E177Q_egfp as compared to RTA_E177D_ suggests that RTA_E177Q_egfp was probably dislocated with slower kinetics than RTA_E177D_. Analysis of glyceraldehyde 3-phosphate dehydrogenase (GAPDH) levels confirmed equal protein loading (Figure 1B, lanes 13–18). Collectively, the data demonstrate that RTA_E177Q_egfp molecules, like RTA_E177D_, gain a single N-linked glycan, and are targeted for retrograde translocation across the ER membrane where RTA species are eventually degraded in proteasome-dependent manner. 

### 2.2. Development of a High-Content Assay to Identify Compounds that Stabilize Ricin A Chain

**Figure 2:**
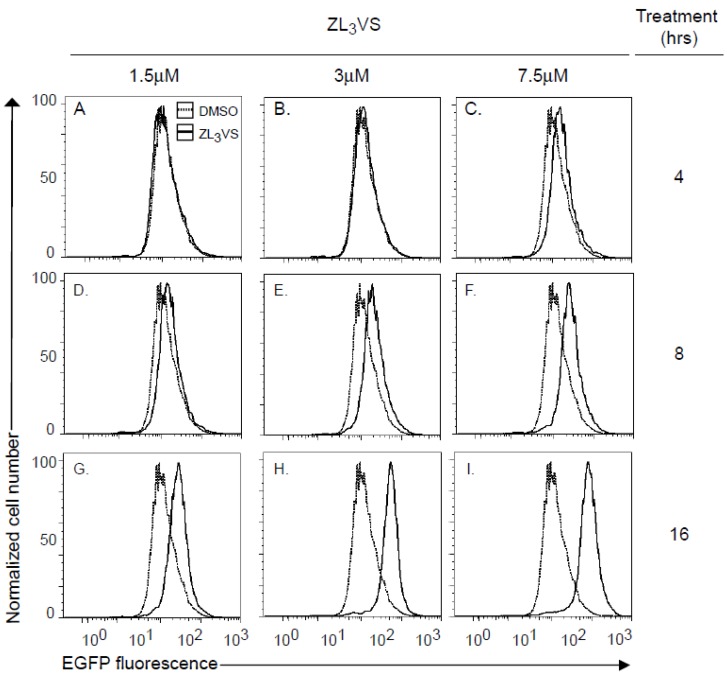
Analysis of RTA_E177Q_egfp stabilization by flow cytometry. U373-RTA_E177Q_egfp cells treated with ZL_3_VS (1.5, 3 or 7.5 µM) for 4 (**A**–**C**), 8 (**D**–**F**) or 16 (**G**–**I**) h were analyzed for EGFP fluorescence intensity using flow cytometry. The data are plotted as normalized cell number *versus* EGFP fluorescence signal.

Using U373-RTA_E177Q_egfp cells, we defined optimal conditions to measure the fluorescence signal from stabilized RTA_E177Q_egfp molecules. U373-RTA_E177Q_egfp cells untreated or treated for 4, 8, or 16 h with 1.5, 3 or 7.5 µM ZL_3_VS were subjected to analysis by flow cytometry (Figure 2). Cells treated with 1.5 or 3 µM ZL_3_VS displayed a significant increase in fluorescence signal only after 16 h of treatment (Figure 2G,H). Yet, the 7.5 µM ZL_3_VS treatment caused a small change in signal after 8 h with a larger increase after 16 h of treatment (Figure 2F,I). The increase in fluorescent signal was exclusively observed in RTA_E177Q_egfp expressing cells and not from ZL_3_VS-treated U373 cells (Supplemental Figure 2). The data indicate that the largest increase in fluorescence signal was observed from cells treated for 16 h under all ZL_3_VS concentrations (Figure 2G–I). Considering cell morphology and toxicity of ZL_3_VS-treated cells (data not shown), the optimal treatment time and concentration of proteasome inhibitor for subsequent assays was determined to be 3 µM ZL_3_VS for 16 h (Figure 2H). 

**Figure 3:**
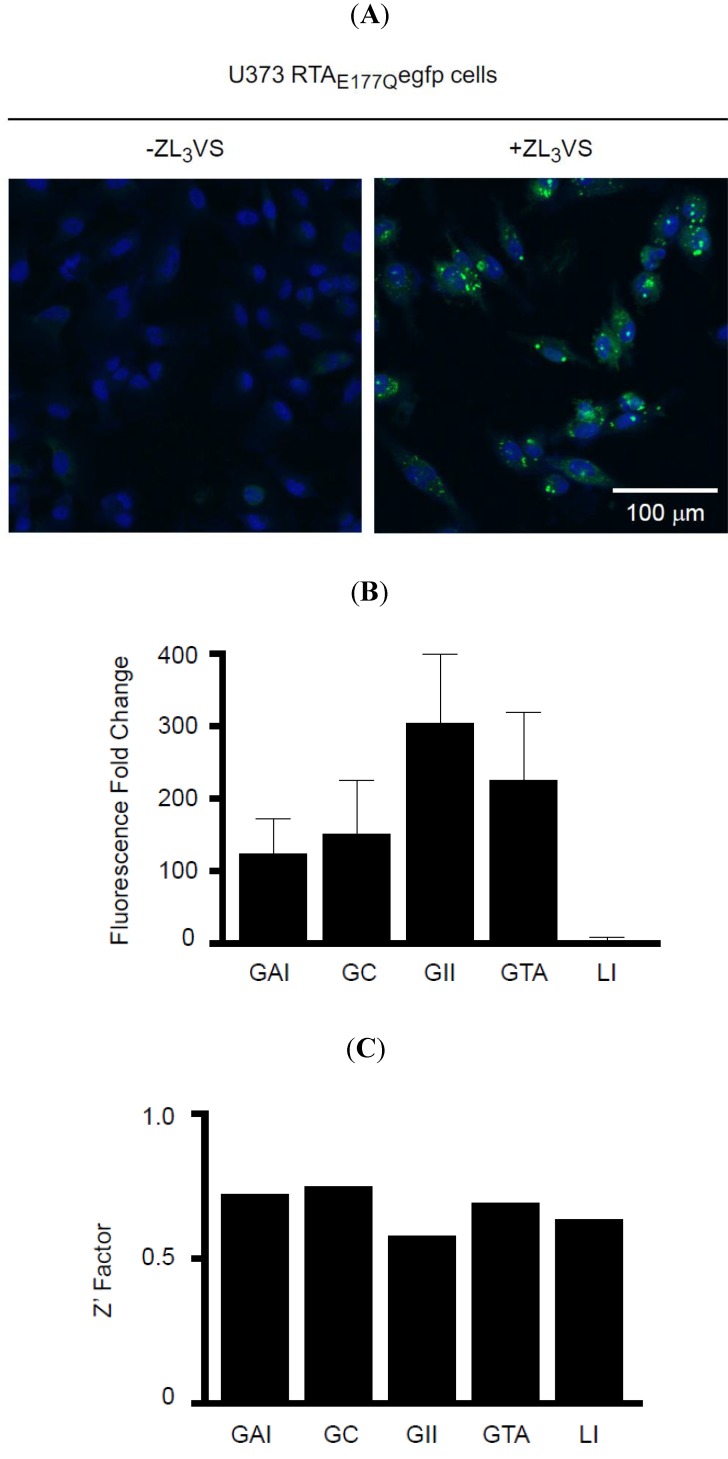
Stabilization of RTA_E177Q_egfp in cells. (**A**) U373 RTA_E177Q_egfp cells treated without or with ZL_3_VS (3 µM, 16h) were fixed, stained with Hoechst reagent, and subjected to confocal fluorescent microscopy. The merged images of the nucleus (blue) and EGFP fluorescent signal from stabilized RTA_E177Q_egfp molecules are shown; (**B**) Fluorescence signal from stabilized RTA_E177Q_egfp molecules was quantified into granule average intensity (GAI), granule count (GC), granule integrated intensity (GII), granule total area (GTA), and Laplacian index (LI). These fluorescence intensity-based values were plotted as fluorescence fold change using DMSO treated cells as background value. The error bars represent calculated fold change from eight independent samples; (**C**) The Z’ factor was determined using the various fluorescent intensity parameters.

An initial experiment to analyze ZL_3_VS-treated U373-RTA_E177Q_egfp cells using a fluorescent plate reader revealed no significant difference in EGFP fluorescence signal upon ZL_3_VS treatment of U373-RTA_E177Q_egfp cells (Supplemental Figure 3). Subsequently, U373 and U373-RTA_E177Q_egfp cells treated with ZL_3_VS were analyzed using plate-scanning confocal fluorescence microscope (Figure 3A). Following proteasome inhibitor treatment, cells were fixed and stained with Hoechst reagent to visualize the nucleus of the cell. Strikingly, U373-RTA_E177Q_egfp cells treated with ZL_3_VS induced distinct peri-nuclear granules (Figure 3A). The fluorescent intensity of these granules were quantified as granule average intensity (GAI), granule count (GC), granule integrated intensity (GII), granule total area (GTA), and Laplacian index (LI) (Figure 3B,C) to determine the most appropriate analysis parameter to determine RTA_E177Q_egfp stability. The comparison of these granularity parameters using non-treated cells as a control demonstrated relative fold change ranging from 3 (LI) to 302 (GII) (Figure 3B). All of the parameters yielded good Z’ factor values > 0.5 with GAI, GC, and GTA generating Z’ factor values > 0.7 (Figure 3C). The largest fold change of GII between untreated or ZL_3_VS treated cells did not produce the highest Z’ Factor (Figure 3B,C). We subsequently selected granule average intensity (GAI) as our analysis parameter which induced a ~124 fold increase over background, a 0.72 Z’ Factor value (Figure 3B,C), and was more consistent among different plates (data not shown). The observation of distinct fluorescent granules upon stabilization of RTA_E177Q_egfp in ZL_3_VS-treated cells provided the basis to perform a high-content screen to identify compounds that stabilize RTA. 

### 2.3. Identification of Hit Compounds from a High-Content Screen that Stabilize RTA_E177Q_egfp Molecules

**Figure 4:**
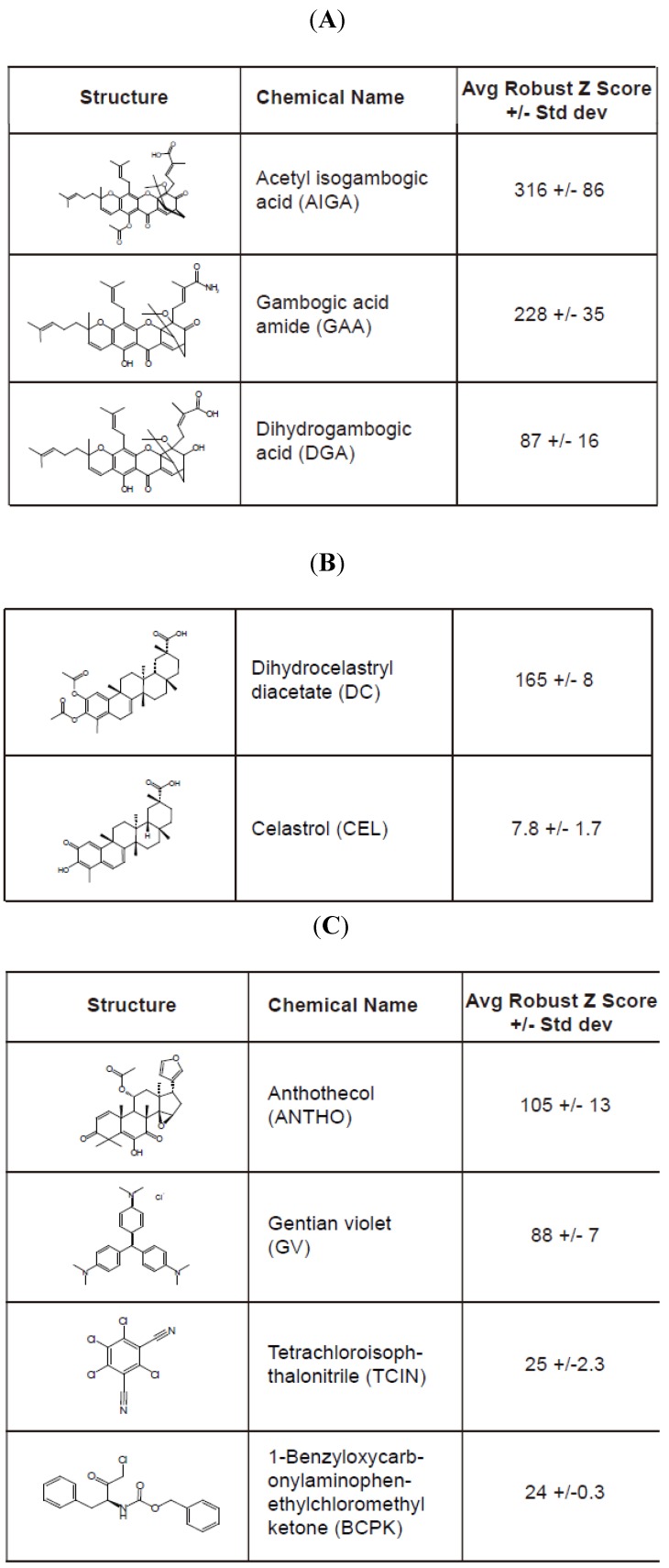
Hit compounds identified from the high-content screen of small chemical library. The hit compounds (**A**–**C**) that induced a reproducible and significant increase of the granule average intensity (GAI) in U373-RTA_E177Q_egfp cells are indicated by their structure, name, and average Robust Z score. The standard deviation value is from three replicates. Two chemically distinct groups of compounds comprised of acetyl isogambogic acid (AIGA), gambogic acid amide (GAA), and dihydrogambogic acid (DGA) (**A**) and dihydrocelastryl diacetate (DC) and celastrol (CEL) (**B**) were identified as hit compounds. Other hit compounds (**C**) include gentian violet (GV), anthothecol (ANTHO), tetrachloroisophthalonitrile (TCIN) and 1-benzyloxycarbonylaminophenethylchloromethyl ketone (BCPK).

We performed a high-content screen using U373-RTA_E177Q_-egfp cells with a bioactive chemical library (2080 compounds, Microsource Discovery Systems, Inc.) using the optimized assay conditions (Material and Methods). In general, U373-RTA_E177Q_egfp cells plated in a 384-well Aurora black clear-bottom microplate were pinned with the compound library for 16 h. Cells were subsequently fixed and analyzed by confocal microscopy to determine the granule average intensity (GAI)/well. The GAI values (in triplicate) were utilized to calculate the Robust Z score for the compound library and eight compounds that produced an average Robust Z score > 20 were considered hit compounds (Figure 4). A large range of values was observed among the hit compounds in which acetyl isogambogic acid (AIGA) yielded a Robust Z score of 316, while 1-benzyloxycarbonylaminophenethylchloromethyl ketone (BCPK) yielded a Robust Z score of 24 (Figure 4). Interestingly, some of the hit compounds clustered into two groups that were structurally related (Figure 4A,B). Note, celastrol (CEL) was selected due to its structurally similarity to dihydrocelastryl diacetate (DC) despite its low Robust Z score. Other structurally similar compounds consisted of acetyl isogambogic acid (AIGA), gambogic acid amide (GAA) and dihydrogambogic acid (DGA). The identification of chemical families that stabilize RTA molecules supports their effectiveness to stabilize RTA molecules. Overall, the RTA_E177Q_egfp-based high-content screen can identify compounds that effectively stabilize RTA polypeptides.

### 2.4. Characterization of Hit Compounds that Stabilize RTA_E177Q_egfp Molecules

**Figure 5:**
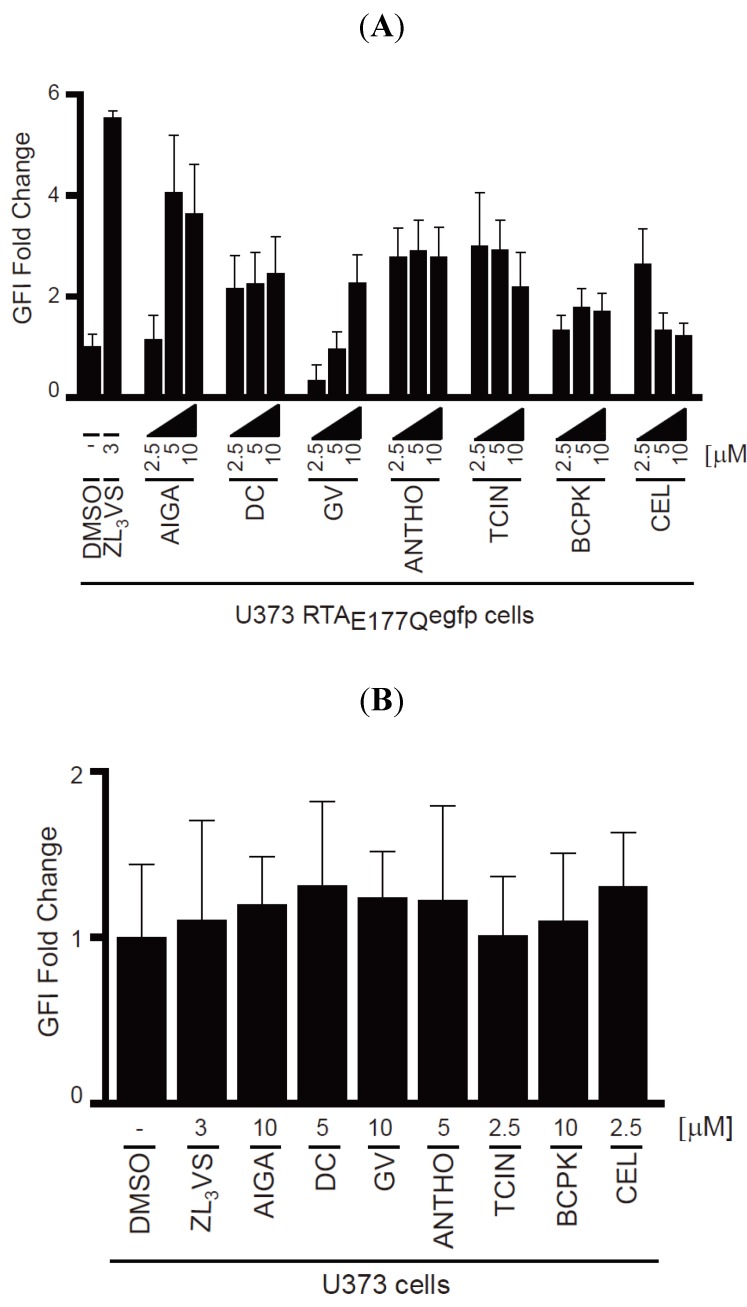
Validation of hit compounds to induce RTA_E177Q_egfp fluorescence intensity. (**A**) U373-RTA_E177Q_egfp cells treated with DMSO, ZL_3_VS (3 µM) or hit compounds (2.5, 5, or 10 µM) for 16 h were subjected to flow cytometry for the analysis of EGFP fluorescence intensity (GFI fold change); (**B**) U373 cells treated with DMSO, ZL_3_VS (3 µM) or hit compounds (indicated concentration) for 16 h were subjected to flow cytometry for the analysis of GFI. The fluorescence signal from the respective treated cells was plotted as GFI fold change utilizing the peak fluorescent signal from cells treated with the various chemicals compared to DMSO treated. Error bars represent the standard deviation of the fluorescent signal from 50% of the peak GFI.

To validate the hit compounds, U373-RTA_E177Q_egfp cells treated with DMSO, 3 µM ZL_3_VS or 2.5, 5 or 10 µM of the hit compounds (Figure 4) for 16 h were assessed by flow cytometry using EGFP fluorescent intensity (Figure 5). The EGFP fold change of U373-RTA_E177Q_egfp cells treated with hit compounds was determined from the peak fluorescent signal of treated cells compared to DMSO-treated cells (Figure 5A). Note, acetyl isogambogic acid was examined as a representative of the gambogic acid family. As expected, ZL_3_VS treatment caused the largest increase in fluorescence signal, quantified as EGFP fluorescent intensity (GFI) fold change, when compared to DMSO treated cells (Figure 5A). Interestingly, the increase in fluorescence signal was concentration dependent for acetyl isogambogic acid (AIGA)- and gentian violet (GV)-treated cells. In contrast, dihydrocelastryl diacetate (DC), anthothecol (ANTHO), tetrachloroisophthalonitrile (TCIN), 1-benzyloxycarbonylaminophenethylchloromethyl ketone (BCPK), and celastrol (CEL) were effective at all concentrations (Figure 5A) suggesting that lower concentrations of these compounds may stabilize RTA molecules. In general, the hit compounds induced a one to four fold increase in fluorescence signal (Figure 5A). These data support the screening assay results that the identified hit compounds induce an increase in fluorescence signal in U373-RTA_E177Q_egfp cells.

To exclude the possibility that the increase in fluorescence signal in U373-RTA_E177Q_egfp cells treated with hit compounds was due to fluorescent properties of the compound, U373 cells treated with the hit compounds were assessed by flow cytometry (Figure 5B). None of the compounds caused a significant increase in fluorescence intensity upon treatment. Thus, the increase in fluorescent intensity observed in treated-U373-RTA_E177Q_egfp cells can be attributed to stabilization of RTA_E177Q_egfp molecules and not the possible fluorescence property of a compound.

To further define the effectiveness of the hit compounds, we determined the half maximal effective concentration (EC_50_) of the hit compounds using U373-RTA_E177Q_egfp cells (Supplemental Figure 4). In general, the EC_50_ values of most compounds ranged from ~1–3 μM with the exception of BCPK and GV whose EC_50_ values were >10 mM. These data provide additional support for the ability of the hit compounds to stabilize RTA polypeptides.

### 2.5. Stabilization of RTA_E177D_ in Cells Treated with Hit Compounds

**Figure 6:**
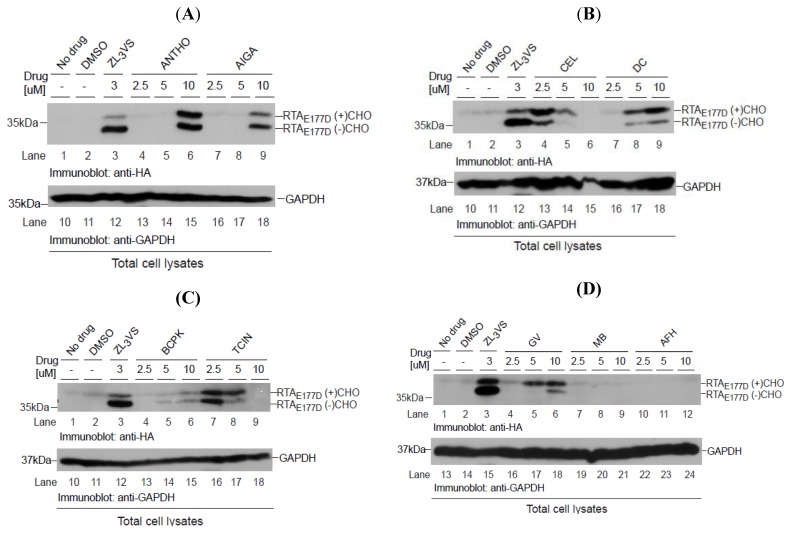
Stabilization of RTA_E177D_ by hit compounds. U373_RTAE177D_ cells treated with DMSO, ZL_3_VS (3 µM) or hit compounds (2.5, 5, and 10 µM) were subjected to immunoblot analysis for RTA_E177D_ (A–C, lanes 1–9; D, lanes 1–12) and GAPDH (A–C, lanes 10–18; D, lanes 13–24). We analyzed the compounds anthothecol (ANTHO), acetyl isogambogic acid (AIGA) (**A**); celastrol (CEL), dihydrocelastryl diacetate (DC) (**B**); 1-benzyloxycarbonylaminophenethylchloromethyl ketone (BCPK), tetrachloroisophthalonitrile (TCIN) (**C**); gentian violet, merbromin (MB), and acriflavinium hydrochloride (AFH) (**D**). RTA polypeptides, GAPDH and molecular weight markers are indicated.

Can the hit compounds stabilize RTA_E177D_ polypeptides? To address this question, U373-RTA_E177D_ cells treated with DMSO, 3 µM ZL_3_VS or 2.5, 5 or 10 µM of hit compounds were subjected to immunoblot analysis (Figure 6). As expected, ZL_3_VS-treated U373-RTA_E177D_ cells accumulated both glycosylated and deglycosylated forms of RTA_E177D_ (Figure 6A–D, lane 3). Strikingly, all of the hit compounds at varying degrees stabilized glycosylated and deglycosylated RTA_E177D_ polypeptides (Figure 6A–C, lanes 4–9 and 6D, lanes 4–6). As controls, U373-RTA_E177D_ cells treated with merbromin (MB) and acriflavinium hydrochloride (AFH), compounds that increased the general nuclear fluorescent signal during the primary high-content screen (data not shown), did not stabilize RTA_E177D_ polypeptides (Figure 6D, lanes 7–12). As a loading control, an immunoblot for GAPDH examined protein levels (Figure 6A–C, lanes 10–18 and 6D, lanes 13–24). Celastrol (CEL) and tetrachloroisophthalonitrile (TCIN) were effective at lower concentrations, yet the higher concentrations may be toxic to cells due to the loss of GAPDH protein (Figure 6A, lanes 4–6, 13–15 and 6C, lanes 7–9, 16–18). Interestingly, celastrol (CV), dihydrocelastryl diacetate (DC) and gentian violet (GV) preferentially stabilized the glycosylated species of RTA_E177D_ (Figure 6B, lanes 4–9 and 6D, lanes 4–6), implying that these compounds interfere with the retrograde translocation step of RTA. Anthothecol (ANTHO), acetyl isogambogic acid (AIGA), 1-Benzyloxycarbonylaminophenethylchloromethyl ketone (BCPK), and tetrachloroisophthalonitrile (TCIN) treatment induced the accumulation of equivalent levels of glycosylated and deglycosylated forms of RTA_E177D_ proteins (Figure 6A, lanes 6 and 9 and 6C, lanes 4–9). The observation of similar amounts of glycosylated and deglycosylated RTA species suggests that these compounds likely target a post-retrograde translocation step. Interestingly, the concentrations required to stabilize RTA_E177D_ were slightly higher than GFP tagged RTA mutant probably due to the faster kinetics of RTA_E177D_ retrograde translocation. In conclusion, the hit compounds were effective reagents to stabilize non-egfp tagged RTA polypeptides.

### 2.6. Inhibition of Ricin Induced Translation Shutdown by Selected Hit Compounds

**Figure 7:**
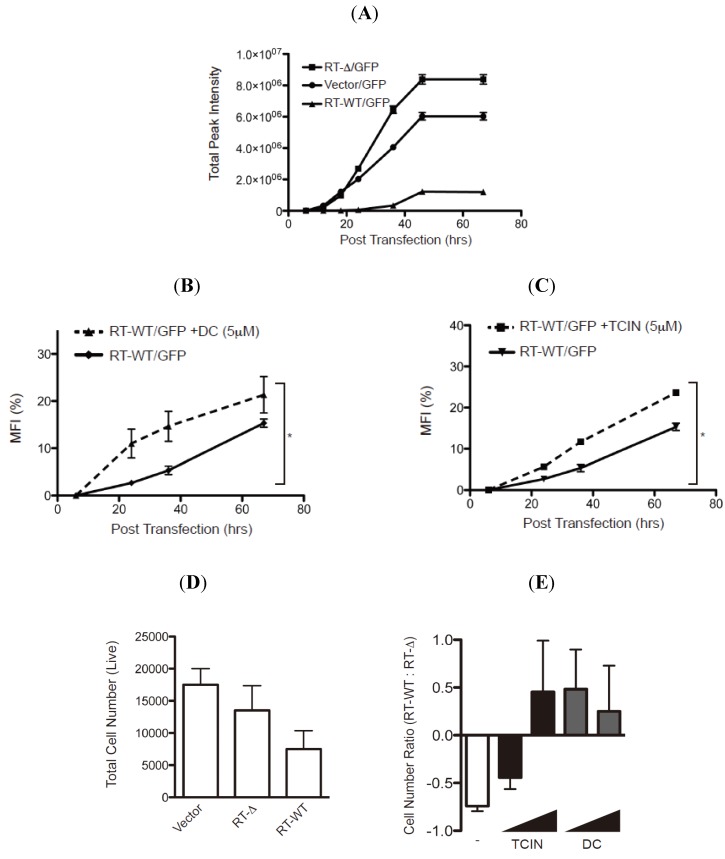
Hit compounds attenuate RTA enzymatic activity. (**A**) HEK-293 cells co-transfected with an GFP-expressing plasmid with vector alone or vectors expressing wild type RTA (RT-WT) or enzymatically inactive mutant RTA-Δ177-181 (RT-Δ) were analyzed for GFP fluorescence over 68 h post-transfection (hpt) using a fluorescence cytometer. The total fluorescence intensity was plotted over time post transfection (h) and the error bars represent signal from six samples. (**B** and **C**) HEK-293 transfected-cells (see above) were treated with dihydrocelastryl diacetate (DC) 6 hpt (**B**) or tetrachloroisophthalonitrile (TCIN) 18 hpt (**C**) followed by analysis of GFP fluorescent signal from six independent samples. The percentage of the mean fluorescent intensity (MFI) from RT-WT transfected cells was calculated using the intensity from RT-Δ transfected cells as 100%. Human fibroblasts transfected with control plasmid, RT-WT, or RT-Δ were untreated (**D**) or treated with TCIN and DC (1 and 5μM) at 18 or 6 hpt (**E**). The total number of viable cells was measured (in quadruple) 48 hpt (**D**). The ratio of viable cells from RT-WT transfected cells to RT-Δ transfected cells from untreated and treated cells was plotted to compare the effect of the respective compound (**E**). The error bars represent the standard deviation between six samples. The data were subjected to a one-tail Student T-test and statistical significance was indicated by an * with *p* values < 0.05.

The stabilization of RTA mutants pre- or post-retrograde translocation by the hit compounds suggests that these reagents may indeed limit the enzymatic activity of wild type ricin to inhibit protein translation. To evaluate the ability of the hit compounds to attenuate enzymatic activity, we established a RTA-activity assay by measuring the GFP fluorescent signal from HEK-293 cells transfected with plasmids encoding GFP with either empty vector, wild type RTA (RT-WT) or an enzymatically inactive RTA molecule lacking residues 177–181 (RT-Δ (Figure 7 and [18]). The GFP fluorescent signal was evaluated for up to 68 h post-transfection (hpt) and as expected a robust increase in total fluorescent signal was observed in the cells transfected with vector alone or RT-Δ (Figure 7A). In contrast, there was only a modest increase in fluorescent intensity in cells transfected with RT-WT validating that enzymatically active RTA inhibits protein synthesis (Figure 7A).

Can the hit compounds limit the enzymatic activity and cytotoxicity of RTA? To address RTA enzymatic activity, HEK-293 cells co-transfected with plasmids encoding GFP and RT-WT or RT-Δ were treated with either dihydrocelastryl diacetate (DC) or tetrachloroisophthalonitrile (TCIN) at either 6 or 18 hpt and evaluated for GFP fluorescence intensity (Figure 7B,C). These experiments examined DC and TCIN because they represent diverse chemical compounds that effectively stabilize RTA (Figure 5 and Figure 6). The percentage mean fluorescent intensity (MFI) from wild type RTA was determined using the fluorescence intensity from the respective RT-Δ expressing cells as 100%. As previously observed (Figure 7A), the MFI from RT-WT expressing cells was >10% MFI at 68 hpt (Figure 7B,C, solid lines). Remarkably, inclusion of dihydrocelastryl diacetate (DC) at 6 hpt or tetrachloroisophthalonitrile (TCIN) at 18 hpt caused a statistical significant increase in MFI in RT-WT expressing cells at all time points post-addition (Figure 7B,C, dashed lines). The results imply that these compounds can attenuate the enzymatic activity of RTA by stabilizing the RTA molecule within the cell.

To examine the effectiveness of TCIN and DC to attenuate RTA-induced cytotoxicity, we performed an initial experiment by determining the number of viable cells followed by the transfection of empty, RT-WT, or RT-Δ expressing plasmids into human fibroblasts (Figure 7D,E). As expected, the most significant decrease in viable cells was from RT-WT-transfected cells. The number of viable cells in drug treated samples was determined as a ratio of viable cells from RT-WT transfected cells to RT-Δ transfected cells (Figure 7E). Hence, untreated cells yielded a negative value (Figure 7E, white bar). Strikingly, the addition of TCIN and DC at increasing concentrations (1 and 5 μM) caused an increase in the RT-WT/ RT-Δ ratio (Figure 7E, black and gray bars). Note, TCIN was more effective at a higher concentration. Collectively, the data supports the paradigm that targeting ER-localized RTA would limit ricin-intoxication. 

## 3. Experimental Section

### 3.1. Cell Lines, cDNA Constructs, Antibodies and Chemicals

Human U373-MG astrocytoma cells stably expressing RTA polypeptides (U373_RTA-E177D_) were generated and maintained as described in DMEM [18,20]. Ricin toxin mutant RTA_E177Q_egfp was generated by the addition of an EGFP molecule to the C-terminus of RTA_E177Q_ using PCR. Gp2-293 cells (Clontech, Palo Alto, CA, USA) were utilized to generate a retrovirus virus with the pLHCX RTA_E177Q_egfp construct and stable human U373-MG cells were selected using hygromycin B (300 µg/mL). U373-RTA_E177Q_egfp cells were then single-cell sorted for low levels of fluorescent signal using a Vantage high-speed cell sorter (Mount Sinai Flow Cytometry Facility). The wild type RTA (RT-WT) and an enzymatically inactive RTA lacking residues 177–181 (RT-Δ) were cloned [18,20]. Anti-GAPDH and anti-calnexin antibodies were purchased from Millipore Corporation and Cell Signaling, respectively. Anti-HA (12CA5) antibodies were purified from hybridoma cells [21]. Carboxybenzyl-Leu-Leu-Leu-vinyl sulfone (ZL_3_VS), Cat #BML-ZW9170, was purchased from Enzo Life Sciences.

### 3.2. Analyzing the Stability of RTA Molecules

U373, U373_RTA-E177D_, and U373-RTA_E177Q_egfp cells treated with ZL_3_VS or respective hit compounds (16 h) were subjected to immunoblot analysis [18,20]. The cells (1 × 10^6^) were lysed in 1× SDS Laemmli sample buffer and the polypeptides were resolved on a 10% SDS-polyacrylamide gel. The proteins were transferred to a PVDF membrane and subjected to a standard protocol for immunoblot analysis. N-linked glycosylation status was analyzed by treating samples with Endoglycosidase H (New England Biolabs, 100 units enzyme/reaction, 37 °C for 1.5 h). 

### 3.3. High-Content Screen

The high-content screen was performed at the Integrated Screening Core of the Experimental Therapeutics Institute at the Icahn School of Medicine at Mount Sinai. The bioactive chemical library (2080 compounds, Microsource Discovery Systems, Inc., Gaylordsville, CT, USA), consisting of FDA-approved drugs, non-FDA approved drugs from other countries and compounds with known bioactive properties, was screened in triplicate. U373-RTA_E177Q_egfp cells (3500 cells/well in 30 μL) were plated in a 384 well Aurora black clear-bottom plate (#1042-11300-S, Brooks Automation, Inc., Chelmsford, MA, USA) overnight at 37 °C. Optimal cell plating density was determined by varying the cell number from 1000 to 10,000 cells/well as was performed in a previous screen [22]. The respective chemical compound (final 5.6 µM) and proteasome inhibitor (ZL_3_VS, final 3.0 µM) were then pinned (17 nL) into the respective well and incubated at 37 °C for 16 h. The wells were permeabilized and fixed using Cytofix/Cytoperm (BD Biosciences) (30 μL, 45 min, 4 °C), washed twice with PBS (30 μL), incubated with Hoechst reagent (2.5 μg/mL final, 45 min, 4 °C), washed with PBS (30 μL), and incubated with PBS (30 μL) followed by sealing the plate with black covers (Cat. No. T-2440-50, ISC BioExpress). Each well was imaged by a Molecular Devices ImageXpress Ultra (IXU) plate-scanning confocal microscope. The IXU plate-scanning confocal microscope collected four fields/well using two lasers: excitation 405 and 488, emission 447/60 and 525/50. Resolution for the images was 2000 scan lines, pixels of 2000 × 2000 and the scanned area was 400 × 400 µm. AcuityXpress Software was used to determine the granule average intensity/cell. Criteria were: nuclei between 7 and 30 µm, 1500 gray levels about local background and granules between 2 and 4 µm and 3000 gray levels about background. Z’ Factor was calculated using the following formula: 1 − ((3σ_C+_ + 3σ_C−_)/(µ_C+_ − µ_C−_)), where σ_C+_ equals the standard deviation of the positive controls, σ_C−_ equals the standard deviation of the negative controls, µ_C+_ equals the mean of the positive controls and µ_C−_ equals the mean of the negative controls [23]. The hit compounds that yielded an average Robust Z score greater than 20 was considered a hit compound [24]. 

### 3.4. Analysis of Fluorescent Signal in U373 RTA_E177Q_egfp Cells with Flow Cytometry

The effectiveness of the proteasome inhibitor ZL_3_VS and the hit compounds to stabilize RTA_E177Q_egfp molecules were examined by flow cytometry analysis. Briefly, U373 or U373-RTA_E177Q_egfp cells treated with DMSO, ZL_3_VS (1.5, 3, and 7.5 µM for 4, 8, and 16 h) or hit compounds (2.5, 5.0, and 10 µM for 16 h) were subjected to flow cytometry using a Beckman Coulter Cytomics FC 500 Flow Cytometer. The data was analyzed using FloJo software from 10,000 events. The EGFP fluorescence intensity curves were plotted based on normalized cell number. Also, the peak fluorescent intensity values were plotted for U373 and U373-RTA_E177Q_egfp cells treated with the hit compounds as EGFP fluorescence intensity (GFI) fold change using DMSO-treated U373-RTA_E177Q_egfp cells or DMSO-treated U373 cells as the background value. The standard deviations for GFI were determined using the signal from 50% of the peak fluorescence value. 

### 3.5. Analysis of Compounds to Block RTA Activity

HEK-293 cells transfected with plasmids encoding for EGFP (1 μg) and wild type RTA or RT-Δ (0.5 μg) were treated with selected hit compounds for up to 60 h post-transfection. The EGFP fluorescent intensity was measured from each sample in a 96 well plate using an Acumen ^e^X3 laser-scanning fluorescence microplate cytometer. The effectiveness of the hit compounds to limit RTA activity was determined as the % mean fluorescent intensity (MFI) using signal from EGFP/RT-Δ transfected cells as background values. The values were subjected to an one-tailed Student T-test (Prism, GraphPad Co., San Diego, CA, USA) for all data points determining the increase in compound treated samples was significance (*p* < 0.05) throughout the time course. Human fibroblasts transfected with empty plasmid (2 μg) and wild type RTA or RT-Δ (2 μg) were treated with selected hit compounds for up to 48 h post-transfection.

## 4. Conclusions

Ricin intoxication requires the ricin A chain to be transported across the ER membrane bilayer to the cytosol where it inhibits protein synthesis. In this study, we utilized a human cell-based high-content screen to identify cell permeable compounds that stabilize the ricin A chain to an intracellular compartment by targeting the transport process across the ER bilayer. Structurally diverse compounds were identified to stabilize the RTA molecules as distinct peri-nuclear species characteristic of ER proteins (Figure 4). These conditions would be expected to cause ricin A chain to aggregate in the ER and eventually be degraded as a misfolded protein. The readout for the high-content screen is an increase in fluorescent signal due to the stabilization of ricin A chain-egfp chimera as fluorescent puncta with a unique size and intensity (Figure 3A). Due to the distinctive nature of the fluorescent “granules”, we were able to uncover effective hit compounds, while excluding chemicals that induce either a general increase in fluorescent signal throughout the cell or specific fluorescent “granules” in non-ER compartments. In addition, toxic compounds would be excluded, as they are unlikely to cause an increase in RTA_E177Q_egfp-induced fluorescent intensity. The robustness of the high-content screen has identified nine effective compounds that stabilize ricin A chains in human cells. More importantly, two compounds attenuated the enzymatic activity and cytotoxicity of ricin A chain (Figure 7) providing further evidence that stabilizing ricin would interfere with its ability to inhibit protein synthesis. 

Compounds that interfere with any step of ricin transport across the ER membrane or its release into the cytosol would stabilize ricin A chain within the cell. We utilized proteasome inhibitor as a positive control because the proteasome plays a critical role in ricin A chain dislocation and may act as a scaffold for the extraction machinery [7]. The wild type holotoxin may utilize the proteasome subunit Rpst5 to ensure proper folding of RTA to block protein synthesis. Even though proteasome inhibitors can stabilize ricin A chain, the primary goal of the high-content screen was to discover compounds that target an early step of retrograde translocation or the retrograde translocation reaction. Consistent with this premise, the identified hit compounds that stabilize RTA molecules by targeting various steps in the retrograde translocation process (Figure 6). The stabilization of glycosylated RTA polypeptides implies that a pre-retrograde translocation step is targeted, while accumulation of a de-glycosylated species is probably affecting a post-retrograde translocation step. Celastrol and dihydrocelastryl diacetate preferentially stabilized glycosylated RTA suggesting that they may stabilize RTA prior to retrograde translocation (Figure 6B). These compounds are isolated from the plant *Tripterygium wilfordii* and have been attributed to increasing chaperone expression, disrupting interactions between Hsp90 and cdc37, and possess proteasome inhibition properties [25,26,27]. Which, if any, of these functions explains the ability of these compounds to stabilize RTA is yet to be determined. Also, gentian violet caused an increase in glycosylated RTA probably from targeting the pre-retrograde translocation reaction. Acetyl isogambogic acid, gambogic acid amide and dihydrogambogic acid are derived from the plant *Garcinia hanburyi* and have also been described to inhibit numerous cell pathways including cell growth (Akt, cSrc, Cdk2 and Cdk4) [28,29,30] and apoptosis (Bcl-2, Bcl-xL and survivin) [31,32,33]. A recent study demonstrates that gambogic acid inhibits the chaperone function of Hsp90, an activity that may explain the inhibition of many cellular processes [34,35]. Interestingly, following retrograde translocation from the ER membrane, RTA must avoid aggregation and refold into a catalytically favorable confirmation to act on its substrate, the ribosome. This process depends on both Hsc70 and Hsp90 so it is intriguing that some of the described functions of our lead compounds correlate with the inhibition of these chaperones [9]. Ideally the most promising compounds would block the toxicity of ricin by interacting with RTA itself or cellular proteins that are necessary for its retrograde translocation from the ER and refolding in the cytoplasm. Treatment with such compounds could limit tissue destruction and multi-organ dysfunction by stabilizing RTA in the ER or in the cytoplasm in a conformation that prevents its catalytic activity.

Drug discovery to identify compounds that inhibit ricin intoxication have utilized various approaches through the analysis of the enzymatic activity of the ricin A chain [36,37,38,39,40,41,42]. Cell-based assays have been used to identify small molecules that have significant abilities to block intracellular transport of both Shiga and ricin toxin [38,40,41]. Additionally, a virtual screening approach identified substrate analogues such as pterines, purines and pyrimidine-based compounds to limit ricin intoxication [37]. The requirement for particular cellular proteins has also been elucidated by cell-based assays. In a recent high-throughput cell-based assay, two lead compounds were discovered that block retrograde trafficking of ricin toxin and protect human pulmonary carcinoma alveolar basal epithelial A549 cells from ricin treatment and mice during a lethal intranasal exposure [43]. Despite the similarity in trafficking pathways utilized by *Pseudomonas* exotoxin and ricin toxin an RNAi screen found an overlap of only 13% in required genes [6]. RNAi screening of *Drosophila melanogaster* S2 cells during treatment with ricin toxin led to the identification of yeast homologues for a protein disulfide isomerase family member and a ubiquitin ligase, archipelago, as necessary for ricin intoxication [44]. These discoveries have provided both unique compounds blocking both aspects of RTA biology and have elucidated required cellular proteins for ricin trafficking. We have identified novel compounds that target the transport of RTA from the ER to the cytoplasm. These compounds were found to stabilize the RTA polypeptides (Figure 6). Remarkably, the compounds inhibited the enzymatic activity and cell-induced toxicity of wild type ricin A chain with statistical significance (Figure 7). Our findings define new targets that would limit ricin intoxication as well as delineate the molecular dynamics of toxin transport.

In conclusion, our reporter cell line was used as the basis of a high-content screen to test a bioactive chemical library, using a combination of confocal microscopy and automated feature extraction to quantify the accumulation of GFP in peri-nuclear puncta (“granules”). This automated assay allowed us to identify compounds that prevent access of ricin toxin A subunit to its substrate by stabilizing RTA in the ER or by blocking its retrograde translocation across the ER membrane. The majority of our lead compounds showed structural similarity and stabilized non-egfp tagged RTA in secondary assays that additionally allowed us to confirm false positives. These compounds will be evaluated further for their ability to stabilize other substrates of ER-associated degradation and possibly other toxins that utilize the ER transport machinery.

## Supplementary Files

Supplementary File 1 Supplementary Information (PDF, 350 KB)
