# Supplementary material for: Novel Class of Potential Therapeutics that Target Ricin Retrograde Translocation
Source: Toxins (Basel). 2013 Dec 23;6(1):33–53. doi: 10.3390/toxins6010033 (PMC3920248; doi:10.3390/toxins6010033)

Supplementary Information

Supplemental Figure 1. ER localization of RTA_{E177Q}egfp. U373, U373-RTA_{E177D}, and U373-RTA_{E177Q}egfp cells treated with DMSO or ZL_3VS (3 μM, 16 h) were undigested or subjected to Endoglycosidase H (EndoH) digestion followed by immunoblot analysis for RTA polypeptides (RTA_{E177Q}egfp: lanes 1–12; RTA_{E177Q}: lanes 13–24; calnexin: lanes 25–35). RTA polypeptides and molecular weight markers are indicated. *: A longer exposure was used to demonstrate expression of RTA_{E177D} polypeptides (lanes 17–18). The glycosylated (+CHO) and deglycosylated (−CHO) RTA species, calnexin, and molecular weight standards are indicated.

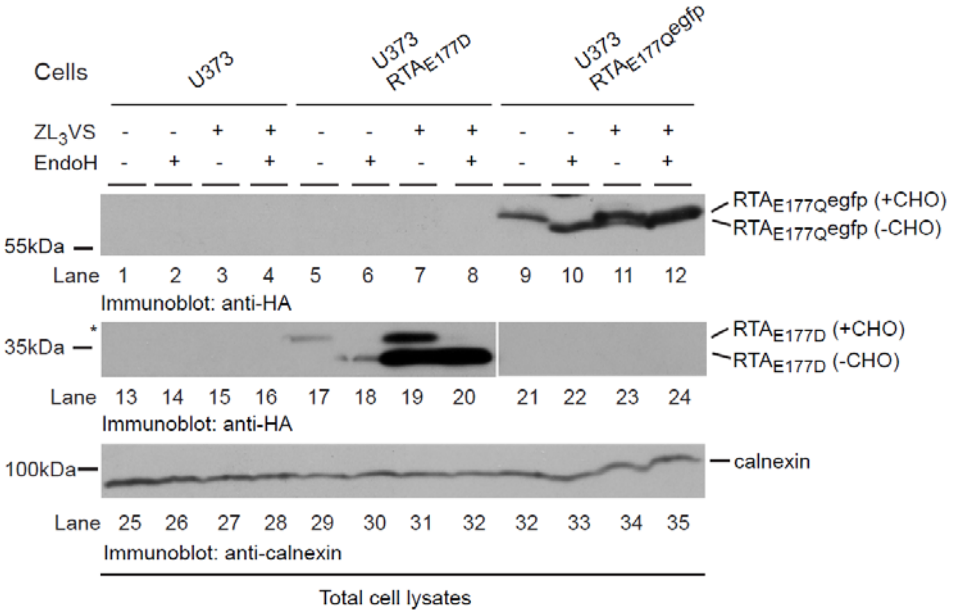

Supplemental Figure 2. Analysis of the fluorescent signal from U373 and U373-RTA_{E177Q}egfp cells by flow cytometry. U373 (**a**) and U373-RTA_{E177Q}egfp (**b**) cells treated with DMSO or ZL_3VS (3 μM, 16 h) were analyzed for EGFP fluorescence using flow cytometry. The EGFP fluorescent intensity was plotted as normalized cell number versus EGFP fluorescence signal.

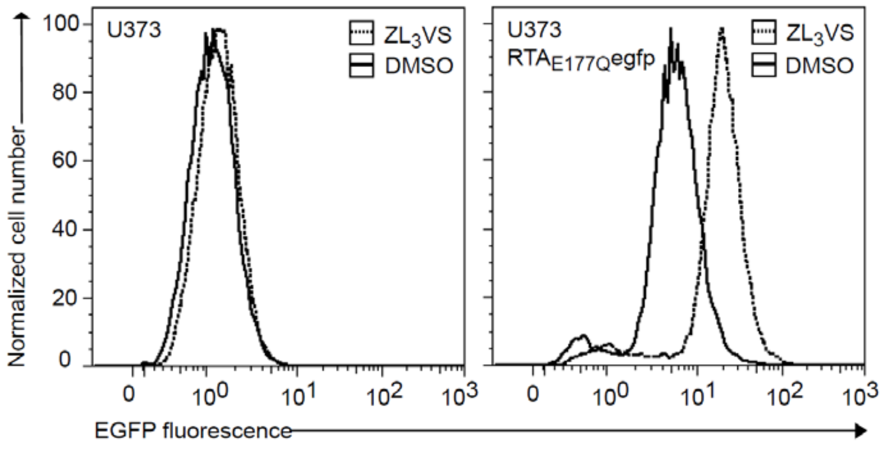

(**a**) (**b**)

Supplemental Figure 3. Analysis of RTA_{E177Q}egfp fluorescent signal using a plate reader. U373 and U373-RTA_{E177Q}egfp (clones 3 and 4) cells seeded at 11,500 (**a**) or 23,000 cells/well (**b**) in a 384 well plate were treated with DMSO (grey bars) or ZL_3VS (3 µM) (black bars) for 16 h, washed 1X with PBS and analyzed for EGFP fluorescence by a Perkin Elmer Envision plate reader. The total EGFP fluorescent signal was plotted for all samples and the error bars represent the standard deviation from ten independent samples.

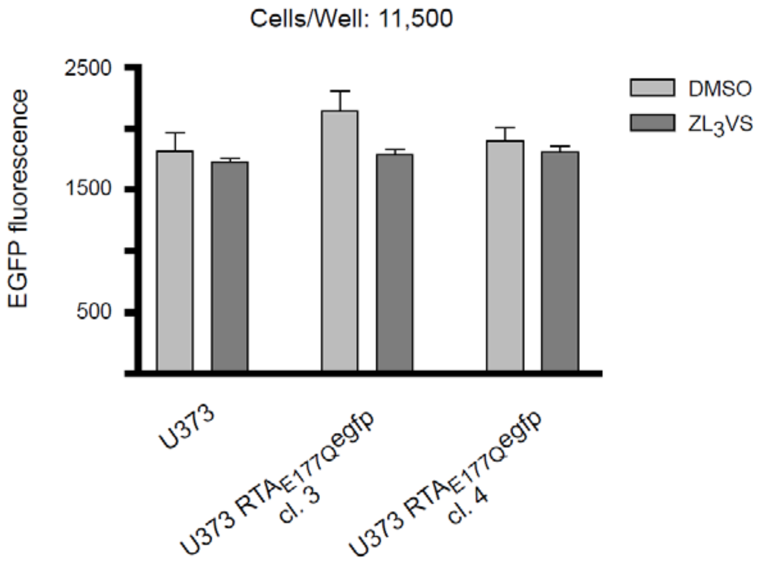

(**a**)

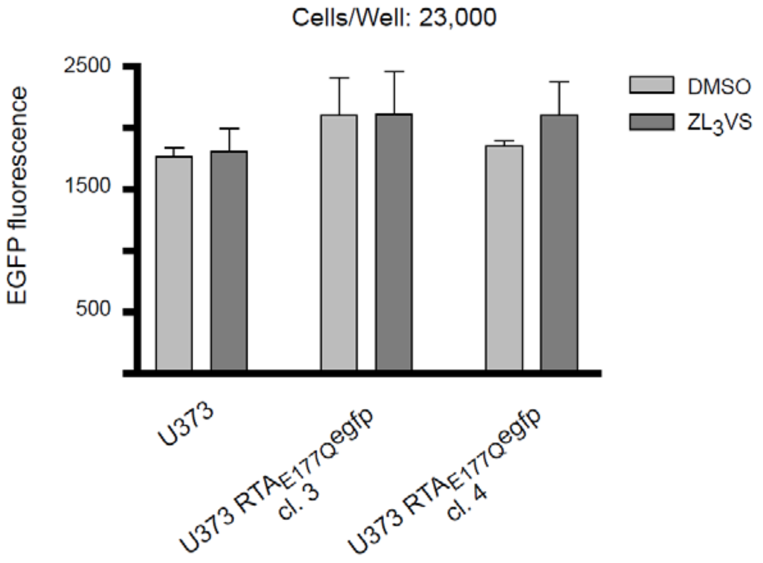

(**b**)

Supplemental Figure 4. Determination of EC_{50} values of the hit compounds. Using the high-content screening conditions, the half maximal effective concentration (EC_{50}) of acetyl isogambogic acid, anthothecol, benzyloxycarbonylaminophenethylchloromethyl ketone, celastrol, dihydrocelastryl diacetate, gentian violet, and tetrachloroisophthalonitrile in U373-RTA_{E177Q}egfp cells was determined by varying the concentration (0–50 mM) of the respective compound using a TecanD300 dispenser. Using the Molecular Devices ImageXpress Ultra (IXU) plate-scanning confocal microscope, the fluorescent multiwavelength cell average intensity (CAI) from duplicate samples was plotted versus compound concentration. The error bars represent the standard deviation between the two samples.

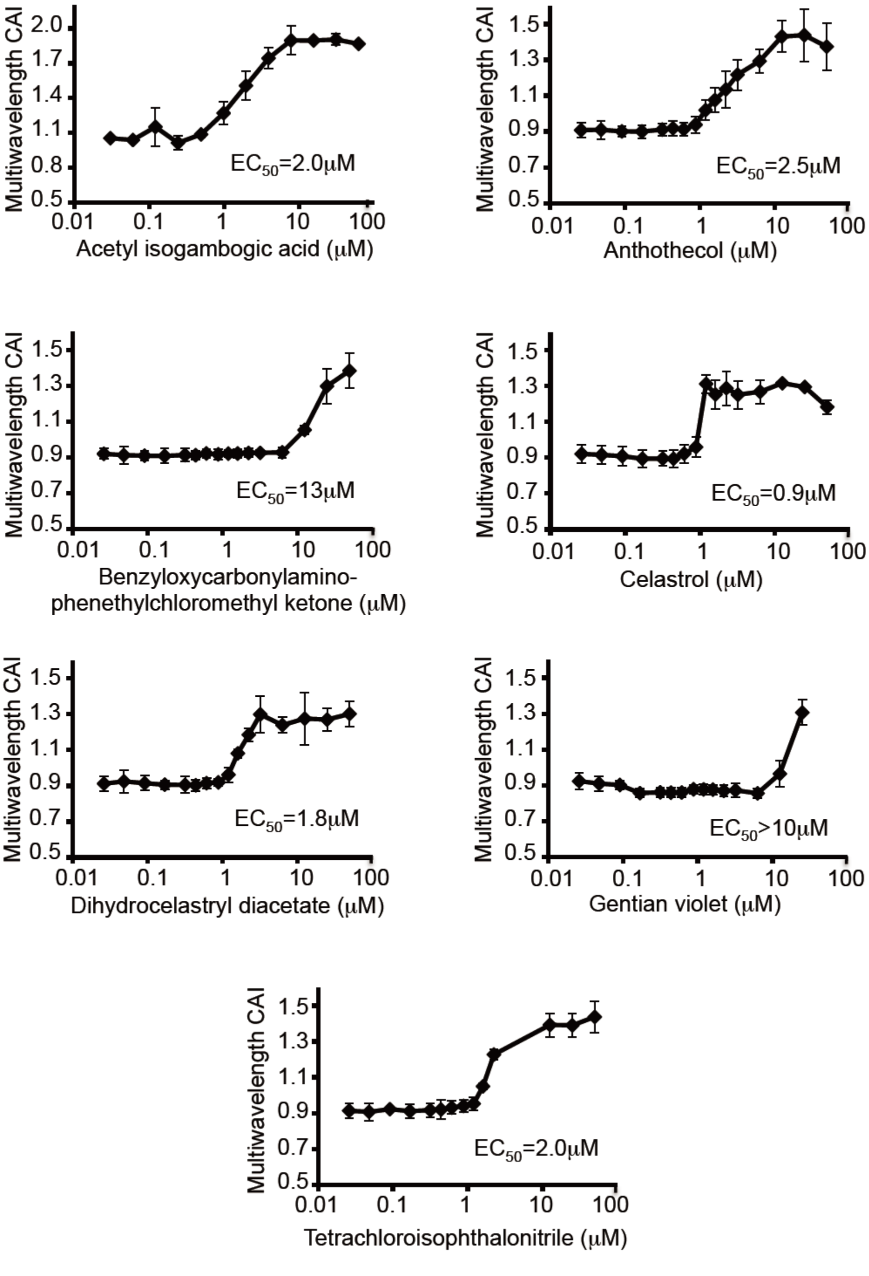

Supplement: Supplementary File 1 — Supplementary Information (PDF, 350 KB) [file toxins-06-00033-s001.pdf]
